# Intertwined roots: linked nitrate and brassinosteroid signaling pathways modulate root system architecture

**DOI:** 10.1093/plphys/kiab413

**Published:** 2021-10-29

**Authors:** Dhineshkumar Thiruppathi

**Affiliations:** Donald Danforth Plant Science Center, Saint Louis, MI 63132, USA

Plants exhibit plasticity in root system architecture (RSA) to adapt to variable nutrient availability. To achieve this, root cells integrate signals from the local external availability of nutrients with their own internal signals to modulate the activity of root uptake systems, thereby matching the nutrient needs of the whole plant. Nitrogen (N) is an important macronutrient modifying RSA, and nitrate (${\text{NO}}_{3}^{–}$) is the major N source for many plants. Regulation of the nitrate uptake and transport systems requires interactions and crosstalk between nitrogen and endogenous hormonal signal transduction pathways, thereby furnishing a rapid and efficient tuning mechanism for optimizing N uptake and root growth in response to N starvation (Krouk, 2016).

RSA modification in response to nitrate deficiency includes morphological changes in lateral and primary roots, such as promotion of lateral and primary root elongation and lateral root number (Gruber et al., 2013). Diverse and delicate molecular events underlie these changes. The primary nitrate response is marked by massive and rapid transcriptional and post-transcriptional regulation of N transporters, assimilation enzymes, and signaling factors (Medici and Krouk, 2014). Depending on ${\text{NO}}_{3}^{–}$ concentration and plant stage, the primary nitrate response branches out to include hormonal signaling e.g. auxin and brassinosteroids (BRs), which appear to feedback on ${\text{NO}}_{3}^{–}$ metabolism, thus blurring the lines between N nutrition and root developmental responses (Krouk, 2016; Jia et al., 2020). While molecular, physiological, and transcriptomic observations demonstrate the nitrate regulation of auxin transport and biosynthesis to stimulate lateral root emergence and lateral and primary root elongation to low-nitrate conditions, such observations for BRs are still emerging.

In this issue of *Plant Physiology*, Song et al. (2021) identify an interaction module composed of CALMODULIN-LIKE-38 (CML38) and PEP1 RECEPTOR 2 (PEPR2) using Arabidopsis (*Arabidopsis thaliana*) as a model plant. They report this module links low-nitrate and BR signals for coordinating root development to prevent quick depletion of N resources. In a previous study, the authors identified diverse root morphology under different nitrate levels and between natural accessions of Arabidopsis (Li et al., 2019). In their new study, they focused on *CML38* as a causative gene responsible for such variation and set out to characterize its protein function in Columbia (Col-0). RT-qPCR and promoter-β-glucuronidase assays confirmed the ${\text{NO}}_{3}^{–}$-dependent expression of *CML38*. Mutation of *CML38* increased primary root length in low- and high-nitrate conditions, and lateral root number in low-nitrate conditions. The authors investigated the role of *CML38* in N uptake, assimilation, and metabolism through biochemical assays and showed increased absorption and assimilation of ${\text{NO}}_{3}^{–}$ in *cml38*, an observation consistent with the upregulation of primary nitrate-response genes, including those encoding various N-transporters and -reductases. They conclude that *CML38* participates in the primary nitrate response in Arabidopsis.

Song et al. (2021) next examined how CML38 mechanistically regulates nitrate assimilation under low-nitrate conditions. They predicted PEPR2 as a potential partner of CML32 by mining public databases and then confirmed their physical interaction through a bimolecular fluorescence complementation assay combined with yeast-two-hybrid and co-immunoprecipitation analyses. PEPR2 is a PEP1 peptide receptor that functions in the perception of Pep peptides and defense responses (Yamaguchi et al., 2010). However, PEPR2 function in N-signaling had not been investigated previously. Through double and triple mutant analyses, the authors confirmed that PEPR2 and CML32 likely work in the same genetic pathway to co-regulate ${\text{NO}}_{3}^{–}$ uptake, assimilation, and metabolism, and root development during the primary nitrate response and that such co-regulation is not related to the canonical Pep1-PEPR2 signaling pathway.

The authors further confirmed that CML38 and PEPR2 play crucial roles in the BR signaling pathway promoted by low nitrate. PEPR2 interacts with BRI1-ASSOCIATED KINASE1 (BAK1), a master regulator of BR signaling (Postel et al., 2010), and mild N deficiency upregulates the transcript levels of BAK1 to activate BR signaling and stimulate root elongation (Jia et al., 2020). Hence, Song et al. (2021) hypothesized the CML32-PEPR2 module as a major point of convergence between N and BRs in the control of N-responsive root development. RT-qPCR and promoter-β-glucuronidase analyses demonstrated the BR-dependent induction of both *CML38* and *PEPR2* in roots. Mutation of *CML32*, *PEPR2*, or both increased primary root length and lateral root number upon BR supply, suggestive of their positive regulation in BR signaling. Combining either low- or high-nitrate conditions with BRs, the authors further confirmed that low-${\text{NO}}_{3}^{–}$ appears to promote BR-inhibited primary root growth. The authors next explored the transcriptome landscapes of the *cml32* and *pepr2* mutant roots exposed to BR plus low-${\text{NO}}_{3}^{–}$ using RNA-seq. They discovered that loss of function of either gene alters common target genes known to function in nitrate and BR signaling pathways.

These results demonstrate CML38 and PEPR2 as important molecular players in the local N signaling pathway that holds many intertwined connections to the root developmental processes and BR signaling. It remains to be studied further how different common target genes of CML38-PEPR2 mechanistically coordinate such connections and whether CML38 and PEPR2 may act in combination to mediate a root-to-shoot-to-root-relay of systemic N-demand signaling (Tabata et al., 2014). Understanding such hierarchical regulation may allow biotechnological engineering of N-efficient crops and improve yield.

*Conflict of interest statement*. None declared.
